# Anti-inflammatory, antibacterial and immunomodulatory treatment in children with symptoms corresponding to the research condition PANS (Pediatric Acute-onset Neuropsychiatric Syndrome): A systematic review

**DOI:** 10.1371/journal.pone.0253844

**Published:** 2021-07-01

**Authors:** Mats Johnson, Stephan Ehlers, Elisabeth Fernell, Parisa Hajjari, Constanze Wartenberg, Susanna M. Wallerstedt

**Affiliations:** 1 Child Neuropsychiatry Centre, Sahlgrenska University Hospital, Gothenburg, Sweden; 2 Gillberg Neuropsychiatry Centre, Sahlgrenska Academy, University of Gothenburg, Gothenburg, Sweden; 3 Regional Knowledge Center for Mental Health, Gothenburg, Sweden; 4 Paediatric Medicine, Kungälv, Sweden; 5 HTA-Centrum, Sahlgrenska University Hospital, Gothenburg, Region Västra Götaland, Sweden; 6 Department of Pharmacology, Sahlgrenska Academy, University of Gothenburg, Gothenburg, Sweden; University of the West Indies at Saint Augustine, TRINIDAD AND TOBAGO

## Abstract

**Objective:**

To assess effects of treatment against a hypothesized neuroinflammation in children with symptoms corresponding to the research condition Pediatric Acute-onset Neuropsychiatric Syndrome (PANS) which is not included in current diagnostic systems.

**Methods:**

Systematic literature searches were performed (1998 to June 2020) in PubMed, Embase, the Cochrane Library, CINAHL, PsycInfo, and HTA databases. Inclusion criteria: patients (P) were children (<18 years) with PANS; intervention (I)/comparison (C) was use of, versus no use of, anti-inflammatory, antibacterial or immunomodulating treatments; outcomes (O) were health-related quality of life (HRQL), level of functioning, symptom change, and complications.

**Results:**

Four randomised controlled trials (RCTs) and three non-RCTs, including 23 to 98 patients, fulfilled the PICO. HRQL was not investigated in any study. Regarding level of functioning, two RCTs investigated antibiotics (penicillin V, azithromycin) and one RCT investigated immunomodulating treatments (intravenous immunoglobulins (IVIG), plasma exchange). Regarding symptoms, two non-RCTs investigated anti-inflammatory treatment (cyclooxygenase (COX) inhibitors, corticosteroids), two RCTs and one non-RCT investigated antibiotics (penicillin V, azithromycin), and two RCTs investigated immunomodulating treatments (IVIG, plasma exchange). Complications, reported in five studies, were consistent with those listed in the summary of products characteristics (SPC). All studies were assessed to have some or major problems regarding directness, the absence of an established diagnosis contributing to clinical diversity in the studied populations. All studies were assessed to have major risk of bias, including selection and detection biases. Due to clinical and methodological diversity, meta-analyses were not performed.

**Conclusion:**

This systematic review reveals very low certainty of evidence of beneficial effects, and moderate certainty of evidence of adverse effects, of anti-inflammatory, antibacterial or immunomodulating treatments in patients with symptoms corresponding to the research condition PANS. Available evidence neither supports nor excludes potential beneficial effects, but supports that such treatment can result in adverse effects.

**Registration:**

PROSPERO (CRD42020155714).

## Introduction

Treatment of children with acute-onset obsessive-compulsive disorder (OCD) or severely restricted food intake combined with other neuropsychiatric symptoms but without a verified neurological/medical disease is controversial. Whilst some researchers in the United States, on the basis of an assumption of an underlying neuroinflammation, recommend anti-inflammatory drugs, antibiotics and immunomodulatory treatment in the clinical management of these patients [1–4], Swedish national guidelines imply that these treatments shall only be provided within the framework of research and development [5].

A rationale to use these treatments was first described in 1994, when a researcher proposed that a subgroup of children with acute-onset OCD, tics and other clinical symptoms suffered from an antineural antibody-mediated dysfunction in the central nervous system [6]. Four years later, a research group in the United States hypothesized that the underlying cause was a streptococcal infection and suggested this condition to be called Pediatric Autoimmune Neuropsychiatric Disorder Associated with Streptococcal infections (PANDAS) [7]. However, a temporal association between a streptococcal infection and the onset of neuropsychiatric symptoms was hard to confirm [8, 9]. It was also difficult to distinguish the onset of tics in the PANDAS group from the non-PANDAS tic disorders. Therefore, in 2012 a group of clinicians and researchers proposed a purely symptom-based entity called Pediatric Acute-onset Neuropsychiatric Syndrome (PANS), based on the clinical descriptions of 400 patients [9]. The criteria included acute onset of OCD or severely restricted food intake in children and adolescents, combined with at least two neuropsychiatric symptoms and in the absence of a verified neurological/medical disease. The condition is currently not included in the fifth diagnostic and statistical manual of mental disorders (DSM-5) or the 10th revision of the International Statistical Classification of Diseases and Related Health Problems (ICD-10), but several cohorts have been described [10–15].

In routine health care, behavioural therapy and psychoactive medications are the established treatment modalities to treat OCD and other psychiatric disorders, antibiotics to treat verified infections, and immunological treatment to treat verified neuroinflammation/autoimmunity reactions. Given the suggestion to use anti-inflammatory, antibacterial or immunomodulatory treatments beyond verified diagnoses [1–4], it may be valuable to assess the certainty of current evidence regarding the benefit-risk balance. Indeed, a previous systematic review, including searches up to October 2017, concluded that the evidence for such treatment had a high risk of bias but no systematic approach was applied to rate the certainty of the evidence [16].

We performed this study to assess the evidence regarding important patient effects of using anti-inflammatory, antibacterial or immunomodulatory treatment, compared with no such treatment, to improve health-related quality of life (HRQL), level of functioning and symptoms in children with symptoms corresponding to the research condition PANS. We also wanted to assess the evidence of complications associated with such treatment.

## Methods

We performed a systematic review according to established routines at the regional health technology assessment (HTA) centre (*HTA-centrum*) in Region Västra Götaland, Sweden. The review was registered with PROSPERO (CRD42020155714). The aim was defined in a PICO (Patients, Intervention, Comparison, Outcome). Patients (P) were children (<18 years) with symptoms corresponding to the research condition of PANS. The intervention (I) was anti-inflammatory, antibacterial or immunomodulating treatments, including cyclooxygenase (COX) inhibitors, glucocorticoids, antibiotics, immunoglobulins, therapeutic plasma exchange, rituximab, and inhibitors of tumour necrosis factor (TNF). The comparison (C) was no anti-inflammatory, antibacterial or immunomodulatory treatment. Outcomes (O) were HRQL according to validated scales, level of functioning including, for example, attendance at school and activities of daily living, symptom change (reported by patients, caregivers and care staff), and complications.

We included both randomised controlled trials (RCTs) and non-randomised controlled trials (non-RCTs). To be able to determine the frequency of complications, we also decided to include case series with >200 patients regarding this outcome. We restricted the search to English, Swedish, Danish, and Norwegian language publications.

### Literature search and study selection

In August 2019, with an update in June 2020, systematic searches were performed in PubMed, Embase, PsycInfo and the Cochrane Library, covering publications from 1998 onwards. Reference lists of relevant articles were scrutinized for additional references. To identify ongoing or completed but not yet published studies, we searched Clinicaltrials.gov in June 2020. Search strategies are provided in S1 Appendix.

Identified abstracts were screened by two persons and those that did not meet the PICO criteria were excluded in a consensus discussion. When there were uncertainties regarding inclusion/exclusion, the full text was retrieved. For articles excluded in consensus, after full-text reading, reasons for exclusions were recorded. The remaining studies were included in the systematic review.

### Data extraction and quality assessment

Data were extracted from the studies by two authors and were subsequently checked by the other authors. Data extraction included the number of individuals in the intervention and control groups, the intervention including dosing, and the results.

The studies were critically appraised by all authors, according to checklists for randomised studies [17] and observational studies [17], respectively, from the Swedish Agency for Health Technology Assessment and Assessment of Social Services (SBU), the national authority for systematic reviews. The assessments, performed according to the routines of the HTA centre, included three domains: directness, risk of bias, and precision. The authors discussed the assessments and categorised each study as having no or minor problems (+), some problems (?), or major problems (–) in each domain. Disagreements were resolved by discussion.

The results of outcomes with >1 RCT were presented in forest plots, using the software Review Manager (RevMan) version 5.4.1 (The Nordic Cochrane Centre, The Cochrane Collaboration, Copenhagen, Denmark). Meta-analyses were not performed; because of clinical and methodological diversity, we considered the studies too heterogenous to provide a meaningful summary estimate. A prior review focusing on antibiotics in PANDAS also refrained from meta-analyses because of heterogeneity [18]. The certainty of evidence, that is, the confidence in the effect estimate, was then assessed using the Grading of Recommendations Assessment, Development and Evaluation (GRADE) [19].

## Results

**Fig 1:**
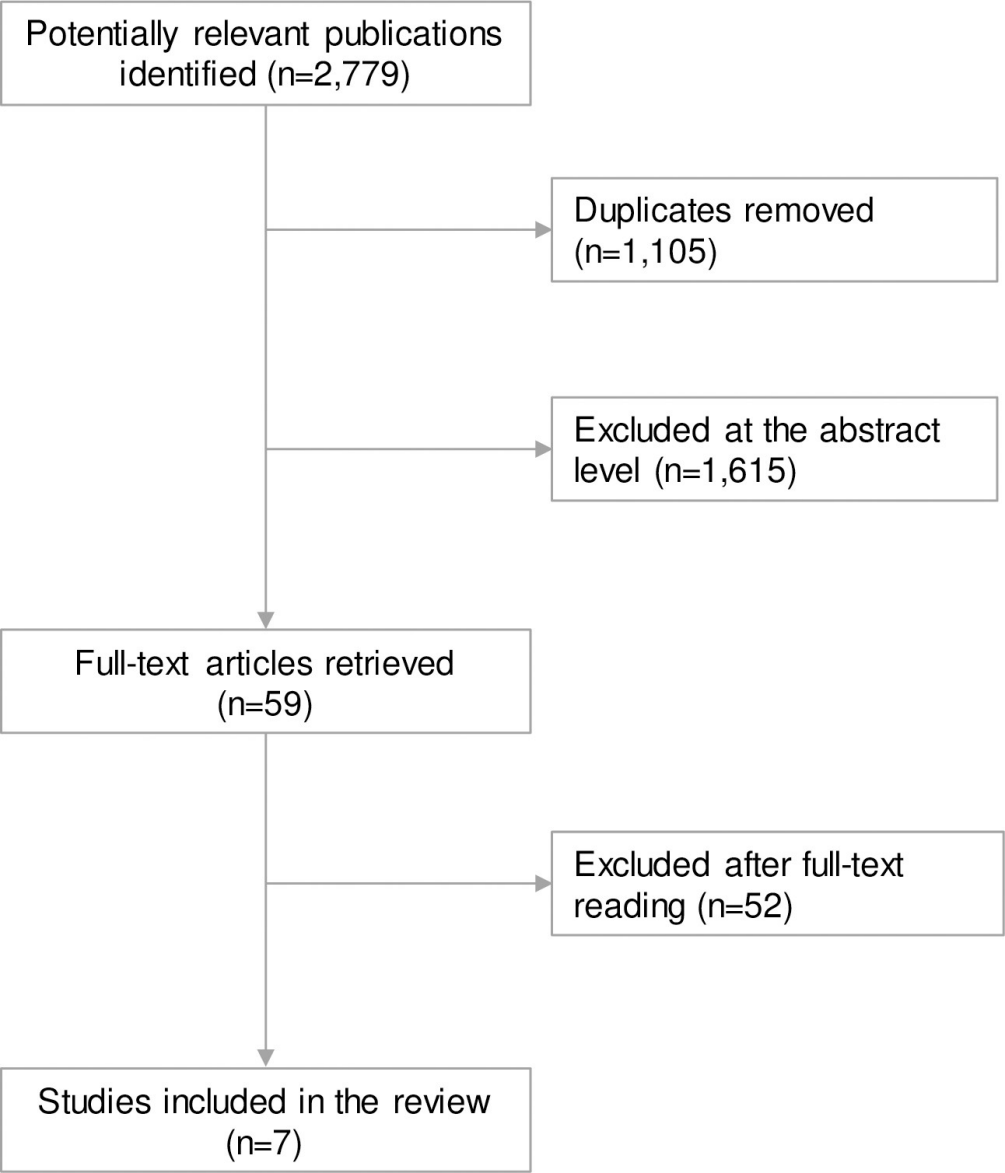
Flowchart of studies included in this systematic review.

After removal of duplicates, the literature search identified 1,674 articles, seven of which fulfilled the PICO of this systematic review (Fig 1). Studies excluded after full-text reading by the authors, as well as the reasons for excluding them, are presented in S2 Appendix.

### Study characteristics

Four RCTs and three non-RCTs were included in the review, including 23 to 98 patients (Table 1). Two studies provided data on anti-inflammatory treatments [20, 21], whereas three studies evaluated antibacterial treatment [22–24] and two studies focused on immunomodulating treatments [25, 26]. These studies investigated effects of COX inhibitors [21], corticosteroids [20], penicillin V [22, 24], azithromycin [23, 24], IVIG [25, 26] and plasma exchange [25]. No studies investigated effects of rituximab or TNF inhibitors. The RCTs had a follow-up of four weeks to four months, and the non-RCTs were both cross-sectional.

**Table 1:** Characteristics of studies fulfilling the PICO criteria.

Author Year	Study design	Patients (n)	I vs C	Results	Comments	Directness*	Risk of bias*	Precision*
			Length of follow-up	Mean (SD)				
Country				I vs C				
Brown et al. 2017 [20] US	Non-RCT, cross-sectional	PANS/PANDAS n = 98	Corticosteroid (85 flares) vs No corticosteroid (318 flares)	Symptoms	102 courses of oral corticosteroids were given to 54 patients	?	-	?
				*Flare duration*				
				All flares: 6.4 (5.0) vs 11.4 (8.6) weeks, P<0.01				
				First flare: 10.3 (5.7) vs 16.5 (9.6), P<0.01				
					Course doses:			
				*Flare duration (weeks) according to corticosteroid course in adjusted model*	1–2 mg/kg orally for 5 days (max dose 60 mg x 2)			
			Data collection September 2012—January 2016					
				Single/relapsing/remitting flare: -3.50 (95% CI: -5.95 to 1.05)				
					Corticosteroids were not prescribed to those in worse psychiatric condition due to concerns about psychiatric adverse effects			
				First flare: -7.49 (95% CI: -13.0 to -1.95)				
				Complications				
				Temporary side effects in 45 out of 102 (44%) courses of steroids: Increase in obsessive-compulsive symptoms (n = 10), anxiety (n = 16), emotional lability/moodiness (n = 12), irritability/agitation (n = 15), sleep disturbance (n = 10), tics (n = 7), aggression (n = 4), urinary symptoms (n = 5), mania (n = 3), sensory amplification (n = 3), hyperactivity (n = 2), hallucinations (n = 2), vision abnormalities (n = 2), behavior regression (n = 2), and flat affect (n = 1).				
					AEs based on review of medical records			
				Of 15 patients who received >5 days or multiple courses of corticosteroids within 1 month, eight (53%) had either weight gain and/or Cushingoid features.				
Brown et al. 2017 [21] US	Non-RCT, cross-sectional	PANS/ PANDAS n = 95	COX inhibitor (43+76 flares) vs No COX inhibitor (271 flares)	Symptoms	Doses: Naproxen 10 mg/kg x 2 (max 500 mg/dose)	?	-	?
				*Flare duration*				
				COX inhibitor early treatment:				
				2.56 fewer weeks (95% CI: -4.68 to -0.43),				
					Ibuprofen 10 mg/kg x 3–4 (max 600 mg/dose)			
				P = 0.018				
				COX inhibitor prophylaxis:	Celecoxib max 50–100 mg x 2			
				4.05 fewer weeks (95% CI: -6.24 to -1.85),	AEs based on review of medical records			
				P<0.0001				
			Data collection September 2012—October 2016	Complications				
					Only patients who tolerated a COX inhibitor for a minimum of 7 days were included, thus excluding patients who may have had side effects in the first week of COX inhibitor therapy			
				19% (11/57 with a COX inhibitor in ≥1 flare) had transient AEs:				
				abdominal pain (n = 5), skin rash (n = 1), bruising (n = 1), proteinuria (n = 3), clinically insignificant transaminitis (n = 1)				
Garvey et al. 1999 [22] US	RCT cross-over	PANDAS	PcV vs Placebo	Level of functioning	Doses: PcV 250 mg x 2 during 4 months	-	-	-
				*CGAS*				
		n = 37		Baseline: 74.27 (10.83) vs 74.27 (10.83)				
			4 months		No systematic reporting of complications			
				4 months: 76.36 (10.31) vs 78.86 (11.67), P = 0.41				
				*NIMH Global Scale*				
				4.48 (1.82) vs 4.33 (1.76), P = 0.39				
				Symptoms				
				*CY-BOCS Obsessions*				
				Baseline: 4.40 (3.93)				
				4 months: 3.27 (4.11) vs 3.96 (5.08), P = 0.16				
				*CY-BOCS Compulsions*				
				Baseline: 3.91 (4.46)				
				4 months: 2.52 (3.90) vs 2.96 (4.93), P = 0.08				
				*YGTSS*				
				Baseline: 15.36 (9.03)				
				4 months: 13.39 (10.65) vs 12.97 (9.49). P = 0.28				
				Complications				
				One case with discoloration of teeth reported by parent				
Murphy et al. 2017 [23] US	RCT	PANS	Azithromycin (n = 17) vs Placebo (n = 14)	Level of functioning	32 individuals randomised, 1 individual (in intervention group) removed from analysis due to food refusal as primary presentation	?	-	-
		n = 32		*CGAS*				
				Baseline: 46.07 (8.37) vs 49.07 (8.38)				
				4 weeks: 53.71 (8.37) vs 52.68 (7.82). P = NS				
			4 weeks	Symptoms				
				CGI-S				
					Dose: Azithromycin 10 mg/kg (max 500 mg/day)			
				Baseline: 5.24 (0.95) vs 5.00 (0.94)				
				4 weeks: 4.06 (0.95) vs 4.93 (0.94), P<0.01				
				*CY-BOCS*				
				Baseline: 29.47 (7.63) vs 28.43 (8.41)	Primary outcome: CGI-S, CYBOCS			
				4 weeks: 20.53 (7.63) vs 23.45 (7.82), P = NS	Secondary outcome: YGTSS			
				*YGTSS*				
				Baseline: 10.88 (8.16) vs 13.21 (8.99)				
					AEs collected by parent/patient report and physical examinations			
				4 weeks: 6.82 (8.16) vs 8.40 (8.34), P = NS				
				Complications				
				*Azithromycin*: Loose stools: 53%. Increased QT in ECG:				
				Four patients with borderline QTc (440–460 ms) at end of week 4 (two of which had borderline QTc at baseline).				
				Within group comparison (QT): P = 0.007				
				Placebo: Loose stools: 7%. Increased QT in ECG:				
				One patient with borderline QTc at end of week 4 (also borderline QTc at baseline).				
				Between group comparison (QT): P = 0.060				
Perlmutter et al. 1999 [25] US	RCT	PANDAS	IVIG/Plasma exchange (n = 9/10) vs Placebo (n = 10)	Level of functioning	30 individuals randomised, 1 individual (in IVIG group) withdrawn owing to non-compliance	?	-	-
				*CGAS*				
		n = 30		*IVIG*:				
				Baseline: 56.0 (9.7) vs 58.3 (10.5)				
				1 month: 67.4 (12.1) vs 59.9 (11.4), P-value NR				
				*Plasma exchange*:	Doses given in the study:			
				Baseline: 56.0 (13.1) vs 58.3 (10.5)				
			1 month	1 month: 73.0 (15.3) vs 59.9 (11.4), P-value NR				
					IVIG 2 g/kg			
					Plasma exchange: 5–6 times Placebo given as sham IVIG			
				Symptoms				
				*CY-BOCS*				
				*IVIG*:	Plasma exchange group not blinded			
				Baseline: 26.7 (5.9) vs 23.0 (13.6)				
				1 month: 14.7 (10.8) vs 22.1 (13.1), P-value NR	After the placebo period, patients were offered active treatment			
				*Plasma exchange*:				
				Baseline: 22.5 (13.4) vs 23.0 (13.6)				
				1 month: 9.5 (10.1) vs 22.2 (13.1), P-value NR.	About 70% of patients had concomitant psychotropic drugs			
				*TSURS*				
				*IVIG*:				
				Baseline: 6.8 (9.2) vs 11.0 (9.5)				
				1 month: 5.5 (7.7) vs 9.7 (9.1), P-value NR				
					Global impairment and severity results reported but scales not described			
				*Plasma exchange*:				
				Baseline: 21.7 (14.7) vs 11.0 (9.5)				
				1 month: 11.0 (9.2) vs 9.7 (9.1), P-value NR				
				Complications				
				*IVIG*: AEs of mild to moderate severity (n = 6):	AEs collected by parent/patient report and physical examinations			
				nausea and vomiting (n = 5), mild to moderately severe headache (n = 3), low-grade fever (n = 4)				
				*Plasma exchange*: Pallor/dizziness/nausea (n = 7), vomiting (n = 2), anxiety (n = 3)				
				*Placebo*: Mild AEs (n = 2): stomachache (n = 2), headache (n = 1)				
Snider et al. 2005 [24] US	Before/ after study	PANDAS	Azithromycin/PcV vs No antibiotic 1 retrospective baseline year compared with 1 prospective study year	Symptoms	Prospectively collected data during 12 months of prophylactic treatment with either PcV or azithromycin, compared with retrospectively collected data from the preceding “baseline” year	?	-	-
				*Number of flares*, *study year vs baseline year*				
		n = 23						
				0.9 (0.5) (azithromycin)/0.5 (0.5) (PcV) vs 2.1 (1.0)/1.8 (0.6), P<0.01				
				Complications				
				AEs were collected but not reported				
					Dose: PcV 250 mg x 2 Azithromycin 250 mg x 2 on one day of the week			
					Significant decrease in streptococcal infections in the study year for both groups			
Williams et al. 2016 [26] US	RCT	PANS/ PANDAS	IVIG (n = 17) vs Placebo (n = 18) 6 weeks	Symptoms	Dose given in the study: IVIG: 2 g/kg	?	-	?
				*CY-BOCS*				
				Baseline: 26.47 (5.14) vs 28.78 (3.98)	Non-responders at 6 weeks (n = 24) were offered IVIG			
		n = 35						
				6 weeks: 20.59 (10.12) vs 25.67 (8.65)				
					All patients received prophylactic antibiotics during the study			
				P = 0.44 (for difference)				
				*CGI-I*				
				6 weeks: 2.88 (1.20) vs 3.53 (1.62), P = 0.12				
				Complications				
				*IVIG*: One patient had a possible allergic reaction which resolved without complication				
					AEs collected by parent/patient report and physical examination			
				Headache (n = 8), sore throat (n = 1), stomach or abdominal discomfort (n = 3), nausea (n = 4), vomiting (n = 3), muscle/bone/joint pain (n = 3), tiredness/fatigue (n = 2), anxiety (n = 2)				
				*Placebo*: Headache (n = 3), sore throat (n = 2), stomach or abdominal discomfort (n = 1), nausea (n = 1), muscle/bone/joint pain (n = 2), tiredness/fatigue (n = 1), anxiety (n = 2)				

*+ = no or minor problems;? = some problems;– = major problems; for directness and risk of bias issues, see S1 Table.

AE = Adverse Event, C = comparison, CALS = Children’s Affective Lability Scale, CGAS = Children’s Global Assessment Scale (score 1–100, with high scores indicating better functioning), CGI-I = Clinical Global Impression Improvement Scale (Score 1–7 where 1 is very much improved and 7 is very much worse), CGI-S = Clinical Global Impression Severity Scale (Score 1–7 where 1 is normal and 7 is the worst), CI = confidence interval, COX = cyclooxygenase, CY-BOCS = Children’s Yale-Brown Obsessive Compulsive Scale (Score 0–40; higher score reflects more symptoms), ECG = echocardiogram, I = intervention, IVIG = Intravenous immunoglobulin, LoF = Level of Functioning, NIMH = National institute of mental health, NR = not reported, NS = not significant (P-value not provided in publication), PANDAS = Pediatric Autoimmune Neuropsychiatric Disorder Associated with Streptococcal infections, PANS = Pediatric Acute-onset Neuropsychiatric Syndrome, PcV = penicillin V, RCT = randomised controlled trial, SCARED = Screen for Childhood Anxiety-Related Emotional Disorders, SD = standard deviation, SE = standard error of the mean, SNAP-IV = Swanson, Nolan, and Pelham-IV Parent Scale, TSURS = Tourette syndrome unified rating scale (Minimum and maximum values are missing), US = United States, YGTSS = Yale Global Tic Severity Scale (Tic severity score 0–50. Higher score = more symptoms) …

All studies had major risk of bias including selection, treatment, detection and reporting biases. None of the studies provided a transparent description of the strategy for evaluating adverse effects. Regarding directness, the studies had major [22] or some [20, 21, 23–26] problems. A major issue was that PANS is not an established diagnosis. As the criteria for the condition have been both restricted and expanded over time [9], the studied populations displayed substantial clinical diversity. Assessments of the risk of bias and directness of the studies are provided in S1 Table.

### Intervention versus control

None of the included studies investigated potential effects of the interventions regarding HRQL.

**Fig 2:**
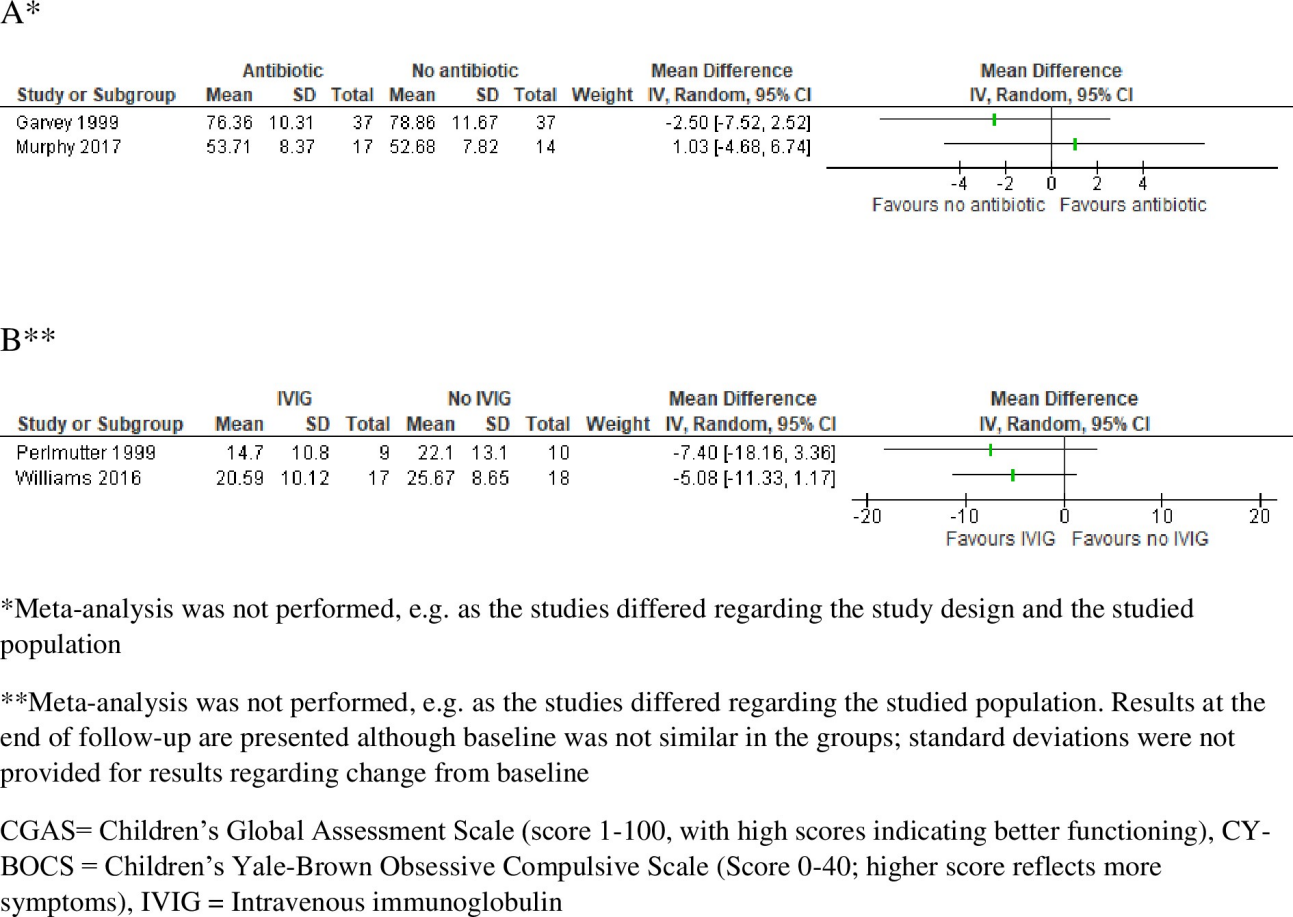
Forest plots for the outcomes *level of functioning* according to CGAS for the comparison antibiotic versus no antibiotic (A), and *symptoms* according to CY-BOCS for the comparisons IVIG versus no IVIG (B). Due to clinical and methodological diversity, in the absence of an established diagnosis and with major risk of bias in all studies (e.g. selection and detection bias), we refrained from pooling the results.

Level of functioning was investigated in two RCTs on antibiotics [22, 23] and in one RCT on intravenous immunoglobulins (IVIG) and plasma exchange [25]. The RCT results regarding potential effects of penicillin V [22] and azithromycin [23], measured by the Children’s Global Assessment Scale (CGAS), are illustrated in a forest plot (Fig 2A). All studies had major risk of bias as, for example, the primary outcome was not clearly defined and reported; there were unclarities regarding the comparability of the randomisation groups; and the blinding could be unmasked by side effects. In the GRADE assessment regarding antibiotics and level of functioning, we downgraded three steps because of risk of bias (uncertainties regarding the comparability of the groups, unblinding due to adverse reactions, multiple comparisons, unclear primary endpoint), indirectness (diagnosis not established, enrichment by excluding prior non-responders) and imprecision (few patients, multiple comparisons). Regarding immunomodulatory treatment, we downgraded three steps because of risk of bias (uncertainties regarding the comparability of the groups, unblinding due to adverse reactions), indirectness (only PANDAS patients, recruited before the research condition was published) and imprecision (few patients). In summary, it is uncertain whether antibiotic or immunomodulatory treatment improves the level of functioning in children with symptoms corresponding to the research condition PANS (GRADE ⊕◯◯◯.

Symptoms were investigated in two cross-sectional studies on anti-inflammatory treatment [20, 21], in two RCTs and one before/after study on antibiotics [22–24], and in two RCTs on immunomodulatory treatment [25, 26]. Confounding by indication was a major issue in the non-RCTs. For instance, corticosteroids were, for most of the study period, not prescribed to those in worse psychiatric condition due to concerns about psychiatric adverse effects. The RCT results regarding potential symptom effects of IVIG, measured by the Children’s Yale-Brown Obsessive Compulsive Scale (CY-BOCS), are illustrated in a forest plot (Fig 2B). In the GRADE assessment regarding potential symptom effects of anti-inflammatory treatment, starting from low certainty evidence as only non-RCTs contributed data, we downgraded one step because of risk of bias and indirectness (confounding by indication and exclusion of patients with more severe symptoms). For antibiotics and immunomodulatory treatment, we downgraded three steps for similar reasons as for level of functioning. In summary, it is uncertain whether anti-inflammatory, antibiotic or immunomodulatory treatment improves symptoms in children with symptoms corresponding to the research condition PANS (GRADE ⊕◯◯◯).

Complications were reported in three RCTs and in two cross-sectional studies. No case series was identified that fulfilled the sample size predefined for inclusion of case series in this review. Adverse effects were reported for anti-inflammatory, antibiotic and immunomodulating treatment. They included abdominal pain and proteinuria for COX inhibitors [21]; increased psychiatric symptoms, weight gain and Cushingoid symptoms for corticosteroids [20]; loose/abnormal stools and prolonged QT for antibiotics [23]; nausea, vomiting, headache, fever, and allergic reactions for IVIG [25, 26]; and vomiting as well as increased anxiety during plasma exchange [25]. As there were relevant between-group differences in adverse events, the observed adverse effects were pharmacologically plausible given the doses provided and concordant with those listed in the summary of products characteristics (SPC), we upgraded the evidence for anti-inflammatory treatment one step, starting from low certainty evidence as only non-RCTs contributed data. For antibiotics as well as pharmacological immunomodulatory treatment, we downgraded one step because of imprecision. For plasma exchange, where regulatory information is less extensive, we downgraded two steps because of imprecision and unblinded treatment. In summary, anti-inflammatory and antibiotic drugs as well as IVIG can probably result in adverse reactions as listed in the SPC (GRADE ⊕⊕⊕◯), and plasma exchange may result in complications (GRADE ⊕⊕◯◯), in children with symptoms corresponding to the research condition PANS.

### Ongoing studies

In all, 28 trials were identified in Clinical Trials, two of which were relevant for our PICO. The first one was a 12-week double-blind RCT, with the aim to investigate the effects of adding benzathine penicillin G to sertraline regarding OCD symptoms and tics (NCT01769027). This study record has not been updated since 2013 and recruitment had not yet started at that time. The second one was a double-blind RCT, with the aim to investigate the effects of the anti-inflammatory agent naproxen regarding OCD symptoms (NCT04015596). This study is recruiting patients as of the last update in November 2020.

## Discussion

This systematic review, based on seven studies with major risk of bias and problems regarding directness and precision, shows that conclusive evidence is largely lacking regarding potential beneficial effects of anti-inflammatory, antibiotic and immunomodulatory treatments for children and adolescents with symptoms corresponding to the research condition PANS. The compiled evidence also indicates that adverse reactions similar to those previously known can probably be expected in this patient group.

None of the studies in this review investigated potential treatment effects regarding HRQL. In the neuropsychiatric field, the Child Health and Illness Profile (CHIP) and Child Health Questionnaire (CHQ) have been shown useful for this purpose [27, 28]. As HRQL reflects the net effect of a treatment, that is, the benefit-risk balance, it could have provided important information. Indeed, we found that the evidence base for beneficial effects was uncertain whereas the treatments could result in adverse reactions. Therefore, it cannot be excluded that the benefit-risk balance may be negative. Given the available evidence, the frequency of adverse reactions could not be determined.

An improved level of functioning would have been a particularly relevant outcome for children meeting the PANS criteria. Indeed, a Swedish study has reported that about two thirds do not attend school for several months [15] and symptoms may include violent outbursts as well as suicidal and homicidal thoughts and gestures [10]. However, none of the studies included in this review showed statistically significant effects regarding this outcome, measured by CGAS. Further, important outcomes as attendance at school and activities of daily living were not used, and future research could have an increased focus on these core aspects of the condition.

Given the results of this review, with major risk of bias in all prevailing studies, efforts in future studies should be made to minimise the risk of bias. Indeed, numerous case reports and case series have shown positive effects of the treatments [16], but adequately designed clinical studies are crucial to contribute to an increased level of evidence. We found that all studies used symptoms as an outcome measure, using a variety of scales covering different aspects of the condition. If/when a diagnosis has been established and verified, it could be of value to define a core outcome measure reflecting the key symptoms of the condition. Efforts have already been made to construct a specific instrument for the patients at issue [29]. Further, as adverse reactions may unblind blinded active treatment, efforts in future RCTs to minimise assessment bias are important. Regarding IVIG, which is associated with, for instance, headache and vomiting, maintaining blinding through the assessments may be particularly difficult. Indeed, in both IVIG trials, vomiting occurred only in the intervention group [25, 26]. In addition, the severity of a reaction may unblind the randomisation group. In the RCTs on IVIG in this review, all reactions of moderate severity occurred in the intervention group [25] or severity was not specified [26]. Unblinding may be particularly problematic when the outcome measures, as in the studies included in this review, are based on subjective assessments. With such outcome measures, the design *per se* also needs thorough consideration to avoid bias. Participants/caregivers who participate in IVIG trials, with efforts and discomfort associated, could be expected to prefer getting active treatment. If the participants/caregivers, as in one of the trials [26], know that they will be guaranteed active treatment only if they do not report symptom relief during the blinded phase, they may be inclined not to report too much improvement, perhaps in particular if they do not experience side effects known to be associated with IVIG. With such an approach they would know for sure to get IVIG at a later stage. Finally, it cannot be excluded that unblinding could have contributed to the somewhat imbalanced baseline characteristics of the comparison groups [25, 26]; the occurrence of adverse reactions for participants in the trial may have suggested what group the subsequent participant would be allocated to.

In future non-RCTs, on the other hand, efforts to minimise confounding by indication will be essential, for instance by propensity score matching [30]. Indeed, COX inhibitors may be avoided in patients with restricted food and/or fluid intake because of their adverse renal effects, and corticosteroids would probably not be prescribed to children with more severe psychiatric symptoms as they may be associated with worsening of such symptoms. Because of this confounding by indication, the compared groups may differ from start and comparisons may not be relevant. In fact, confounding by indication is frequent in observational studies [31, 32], a problem which diminishes their potential to contribute to the evidence base.

The main strength of this systematic review is that it gives an overview of the currently available evidence of treatments of controversy for children with symptoms corresponding to PANS, a research condition that can imply a considerable caregiver burden [33]. Limitations include that few studies fulfilled our PICO criteria and that all studies had a considerable risk of bias. In addition, the immaturity of the condition, not being an established diagnosis, may contribute to difficulties to evaluate potential treatment effects; the patient population at issue may be hard to distinguish from other OCD patients. The criteria have been modified over the years and indirectness may continue to be a major problem for research on treatment effects until a diagnosis with clear boundaries can be defined and verified. Indeed, it has not yet been possible to identify reliable biomarkers for PANS [8, 34, 35]. In the absence of an established diagnosis, studied populations may not be sufficiently homogenous to assume that similar treatment strategies would be applicable. In this review, we focus on treatment against a suspected underlying neuroinflammation, but it needs to be acknowledged that neither the natural course of the combination of symptoms included in the suggested PANS/PANDAS criteria nor the benefit-risk balance of other treatment strategies, including behaviour therapy and psychoactive medications, have been established.

To conclude, this systematic review suggests that current evidence regarding the benefit-risk balance may not be positive for providing treatment against a hypothesised underlying neuroinflammation/autoimmunity to children with acute-onset OCD or severely restricted food intake, combined with other neuropsychiatric symptoms but without a verified neurological/medical disease. The evidence base is too uncertain to support or exclude beneficial effects, whereas there is moderate certainty evidence that such treatment can result in adverse effects. It may be noted that this evidence situation has similarities with the one regarding treatment of juvenile idiopathic arthritis, where reviews have shown that the level of evidence is mostly “very low” or “low” for COX inhibitors, corticosteroids and other currently available medications [36].

## Supporting information

S1 Checklist (DOC)

S1 Table Aspects of directness and risk of bias identified during the assessment process contributing to the study being categorised as having no/minor (+), some (?), or major (-) problems. (DOCX)

S1 Appendix Search strategies. (DOCX)

S2 Appendix Studies excluded after full-text reading, as well as the reason for excluding them. (DOCX)
