# Supplementary material for: Anti-inflammatory, antibacterial and immunomodulatory treatment in children with symptoms corresponding to the research condition PANS (Pediatric Acute-onset Neuropsychiatric Syndrome): A systematic review
Source: PLoS One. 2021 Jul 1;16(7):e0253844. doi: 10.1371/journal.pone.0253844 (PMC8248649; doi:10.1371/journal.pone.0253844)
Supplement: S1 Table — (DOCX) [file pone.0253844.s002.docx]

**S1 Table.** Aspects of directness and risk of bias identified during the assessment process contributing to the study being categorised as having no/minor (+), some (?), or major (-) problems.

| Author Year  Country | Study design | Directness | | Risk of bias | |
| --- | --- | --- | --- | --- | --- |
|  |  | +/?/- | Problems | +/?/- | Problems |
| Brown et al. 2017  US | Non-RCT, cross-sectional | ? | PANS/PANDAS*  Acute onset of disease not required, only ≥1 acute onset flare  Only 98 out of 165 patients meeting symptom criteria included:   - Patients with more severe symptoms excluded - Patients with inadequate data excluded - Unclarity regarding number of flares excluded | - | Not comparable groups:   - Confounding by indication: corticosteroids not prescribed to those in worse psychiatric condition due to concerns about psychiatric adverse effects - Higher PANS Global Impairment Score in intervention group - Contingency plans for additional supportive therapy and personnel when corticosteroids were provided |
| Brown et al. 2017  US | Non-RCT, cross-sectional | ? | PANS/PANDAS*  Acute onset of disease not required, only ≥1 acute onset flare  Only 95 out of 200 patients meeting symptom criteria included:   - Patients with more severe symptoms excluded - Patients with inadequate data excluded - 129 of 519 flares included | - | Not comparable groups:   - Confounding by indication: COX inhibitors may be avoided in patients with restricted food and/or fluid intake because of their adverse renal effects - Characteristics differ between compared groups |
| Garvey et al. 1999  US | RCT cross-over | - | PANDAS*  Patients recruited before the criteria of PANDAS were proposed  Study population with a high level of functioning: 28/37 attended school throughout the study, 9/39 attended ≥6/8 months  Provided dose inadequate to maintain continued  streptococcal prophylaxis | - | Characteristics of compared groups not reported, e.g. regarding concomitant psychotropic medication  Primary outcome not defined  First 2 study years: Screening for throat infection every month, treated with antibiotics if positive culture, irrespective of symptoms, in both randomisation groups |
| Murphy et al. 2017  US | RCT | ? | PANS*  One intervention patient removed from analysis due to food refusal as primary presentation  Previous non-responders were excluded | - | Randomisation procedure not described, randomisation groups not apparently well balanced regarding duration of illness, use of SSRI, and infectious triggers  Unclarity regarding primary outcome: CY-BOCS or CGI-S  Multiple statistical tests not corrected for |
| Perlmutter et al. 1999  US | RCT | ? | PANDAS*  Patients recruited before the criteria of PANDAS were proposed  Recruitment process may select specifically motivated patients | - | Some clinical imbalance in characteristics of compared groups  Detection bias:   - Plasmapheresis not blinded - IVIG blinded, but side effects contribute to unblinded outcome assessments - If randomised to placebo (which could be revealed by lack of side effects) and reporting no symptom improvement, open treatment with IVIG or plasmapheresis was offered   Primary outcome not defined |
| Snider et al. 2005  US | Before/ after study | ? | PANDAS*  Unclear disease severity | - | Detection bias: data were obtained retrospectively for the control period and prospectively for the intervention period  Primary outcome not defined  Results presented at the aggregated level (number of neuropsychiatric exacerbations). No results provided for the rating scales used |
| Williams et al. 2016  US | RCT | ? | PANS/PANDAS*  Prophylactic antibiotics prescribed to all participants | - | Some clinical imbalance in characteristics of compared groups  Detection bias:   - IVIG blinded, but side effects contribute to unblinded outcome assessments - IVIG was offered open label to non-responders after the blinded phase (lack of side effects potentially revealing randomisation to placebo) |

*Condition not an established diagnosis in DSM-5 or ICD-10, proposed criteria changed over the years

COX = cyclooxygenase, DSM-5 = fifth diagnostic and statistical manual of mental disorders, ICD-10 = 10th revision of the International Statistical Classification of Diseases and Related Health Problems, IVIG = Intravenous immunoglobulin, PANDAS = Pediatric Autoimmune Neuropsychiatric Disorder Associated with Streptococcal infections, PANS = Pediatric Acute-onset Neuropsychiatric Syndrome, SSRI = selective serotonin reuptake inhibitor, US = United States
