# Supplementary material for: Anti-inflammatory, antibacterial and immunomodulatory treatment in children with symptoms corresponding to the research condition PANS (Pediatric Acute-onset Neuropsychiatric Syndrome): A systematic review
Source: PLoS One. 2021 Jul 1;16(7):e0253844. doi: 10.1371/journal.pone.0253844 (PMC8248649; doi:10.1371/journal.pone.0253844)
Supplement: S1 Appendix — (DOCX) [file pone.0253844.s003.docx]

**S1 Appendix.** Search strategies

**Database:** PubMed

**Date:** 22 Aug 2019
**No. of results:** 911
**Search updated:** 3 June 2020, 75 results (Legacy PubMed)

| **Search** | **Query** | **Items found** |
| --- | --- | --- |
| [**#10**](https://www.ncbi.nlm.nih.gov/pubmed/advanced) | **Search #6 NOT #7 Filters: Publication date from 1998/01/01; Swedish; Norwegian; English; Danish** | **911** |
| [#9](https://www.ncbi.nlm.nih.gov/pubmed/advanced) | Search #6 NOT #7 Filters: Swedish; Norwegian; English; Danish | 1,022 |
| [#8](https://www.ncbi.nlm.nih.gov/pubmed/advanced) | Search #6 NOT #7 | 1,090 |
| [#7](https://www.ncbi.nlm.nih.gov/pubmed/advanced) | Search Editorial[ptyp] OR Letter[ptyp] OR Comment[ptyp] | [1,753,875](https://www.ncbi.nlm.nih.gov/pubmed/?cmd=HistorySearch&querykey=7) |
| [#6](https://www.ncbi.nlm.nih.gov/pubmed/advanced) | Search #1 NOT #5 | 1,139 |
| [#5](https://www.ncbi.nlm.nih.gov/pubmed/advanced) | Search #2 OR #3 OR #4 | [4,930,918](https://www.ncbi.nlm.nih.gov/pubmed/?cmd=HistorySearch&querykey=5) |
| [#4](https://www.ncbi.nlm.nih.gov/pubmed/advanced) | Search ((animals[mh]) NOT (animals[mh] AND humans[mh])) | [4,611,370](https://www.ncbi.nlm.nih.gov/pubmed/?cmd=HistorySearch&querykey=4) |
| [#3](https://www.ncbi.nlm.nih.gov/pubmed/advanced) | Search animal[ti] OR animals[ti] OR rat[ti] OR rats[ti] OR mouse[ti] OR mice[ti] OR rodent[ti] OR rodents[ti] OR dog[ti] OR dogs[ti] OR cat[ti] OR cats[ti] OR hamster[ti] OR hamsters[ti] OR rabbit[ti] OR rabbits[ti] OR swine[ti] OR murine[ti] | [1,830,757](https://www.ncbi.nlm.nih.gov/pubmed/?cmd=HistorySearch&querykey=3) |
| [#2](https://www.ncbi.nlm.nih.gov/pubmed/advanced) | Search giant panda*[tiab] OR red panda*[tiab] OR greater panda*[tiab] | [673](https://www.ncbi.nlm.nih.gov/pubmed/?cmd=HistorySearch&querykey=2) |
| [#1](https://www.ncbi.nlm.nih.gov/pubmed/advanced) | Search PANDAS[tiab] OR PANS[tiab] OR ((pediatric OR pediatric-onset OR paediatric-onset OR childhood OR childhood-onset OR children OR child) AND (autoimmune OR acute OR acute-onset OR recent-onset) AND (neuropsychiatric OR neuro-psychiatric)) | 1,623 |

**Database: Embase**1974 to 2019 August 21 (OvidSP)

**Date:** 22 Aug 2019

**No. of results:** 1074
**Search updated:** 3 June 2020, 69 results

| **#** | **Searches** | **Results** |
| --- | --- | --- |
| 1 | (PANS or PANDAS).ab,kw,ti. | 1,535 |
| 2 | (p?ediatric or p?ediatric-onset or childhood or childhood-onset or children or child).mp. [mp=title, abstract, heading word, drug trade name, original title, device manufacturer, drug manufacturer, device trade name, keyword, floating subheading word, candidate term word] | 2,602,306 |
| 3 | (autoimmune or acute or acute-onset or recent-onset).mp. [mp=title, abstract, heading word, drug trade name, original title, device manufacturer, drug manufacturer, device trade name, keyword, floating subheading word, candidate term word] | 1,943,825 |
| 4 | (neuropsychiatric or neuro-psychiatric).mp. [mp=title, abstract, heading word, drug trade name, original title, device manufacturer, drug manufacturer, device trade name, keyword, floating subheading word, candidate term word] | 43,579 |
| 5 | 2 and 3 and 4 | 1,159 |
| 6 | 1 or 5 | 2,312 |
| 7 | (giant panda$ or red panda$ or greater panda$).ab,kw,ti. | 659 |
| 8 | (animal or animals or rat or rats or mouse or mice or rodent or rodents or dog or dogs or cat or cats or hamster or hamsters or rabbit or rabbits or swine or murine).ti. | 1,942,617 |
| 9 | (animal not (animal and human)).sh. | 1,044,840 |
| 10 | 7 or 8 or 9 | 2,753,087 |
| 11 | 6 not 10 | 1,882 |
| 12 | limit 11 to yr="1998 -Current" | 1,720 |
| 13 | limit 12 to ((embase or medline) and (article or article in press or conference paper or note or "review")) | 1,177 |
| **14** | **limit 13 to (danish or english or norwegian or swedish)** | **1,074** |

**Database:** PsycInfo (EBSCOhost)

**Date:** 22 Aug 2019

**No. of results:** 522
**Search updated:** 3 June 2020, 27 results

| **#** | **Query** | **Result** |
| --- | --- | --- |
| **S9** | **Limit S8 to Academic journals** | **522** |
| S8 | S1 OR S5 Limit: Publication date: 19980101-20191231 | 583 |
| S7 | S1 OR S5 Limit: English | 648 |
| S6 | S1 OR S5 | 682 |
| S5 | S2 AND S3 AND S4 | 499 |
| S4 | neuropsychiatric or neuro-psychiatric | 39,543 |
| S3 | autoimmune or acute or acute-onset or recent-onset | 99,894 |
| S2 | p#ediatric or p#ediatric-onset or childhood or childhood-onset or children or child | 845,644 |
| S1 | TI ( "pans" OR "pandas" ) OR AB ( "pans" OR "pandas" ) | 365 |

**Database:** The Cochrane Library

**Date:** 22 Aug 2019

**No. of results: 95**

*Cochrane reviews 1*

*Trials 94***Search updated:** 3 June 2020, 6 results *(all Trials)*

| **ID** | **Search** | **Hits** |
| --- | --- | --- |
| #1 | ("pans" OR "pandas"):ti,ab,kw | 63 |
| #2 | (p*ediatric or p*ediatric-onset or childhood or childhood-onset or children or child):ti,ab,kw | 142,848 |
| #3 | (autoimmune or acute or acute-onset or recent-onset):ti,ab,kw | 131,348 |
| #4 | (neuropsychiatric or neuro-psychiatric):ti,ab,kw | 2,931 |
| #5 | #2 AND #3 AND #4 | 51 |
| **#6** | **#1 OR #5** | **95** |

The web-sites of **SBU** and **Folkehelseinstituttet** were visited

23 Aug 2019

Nothing relevant to the question at issue was found

A comprehensive review of reference lists brought no new records

**Searches in clinicaltrials.gov**

**A search in Clinicaltrials.gov (June 9, 2020) using the search terms** AREA[ConditionSearch]:

*PANDAS OR ((pediatric OR pediatric-onset OR paediatric-onset OR childhood OR childhood-onset OR children OR child) AND (autoimmune OR acute OR acute-onset OR recent-onset) AND (neuropsychiatric OR neuro-psychiatric))* **identified 28 studies.**
