# Supplementary material for: Anti-inflammatory, antibacterial and immunomodulatory treatment in children with symptoms corresponding to the research condition PANS (Pediatric Acute-onset Neuropsychiatric Syndrome): A systematic review
Source: PLoS One. 2021 Jul 1;16(7):e0253844. doi: 10.1371/journal.pone.0253844 (PMC8248649; doi:10.1371/journal.pone.0253844)
Supplement: S2 Appendix — (DOCX) [file pone.0253844.s004.docx]

**S2 Appendix.** Studies excluded after full-text reading, as well as the reason for excluding them.

C = comparison, I = intervention, O = outcome, OCD = obsessive-compulsive disorder, P = patients, PANDAS = Pediatric Autoimmune Neuropsychiatric Disorder Associated with Streptococcal infections, PANS = Pediatric Acute-onset Neuropsychiatric Syndrome

| **Author, year** | **Reason for exclusion** |
| --- | --- |
| Bejerot 2013 | Wrong design (review) |
| Blackburn 2018 | Wrong design (review) |
| Burchi 2018 | Wrong design (systematic review) |
| Calaprice 2017 | Wrong I (no treatment) |
| Calaprice 2018 | Wrong P (2-38 years) |
| Celik 2016 | Wrong I (no treatment) |
| Cocuzza 2019 | Wrong I (no treatment) |
| Cooperstock 2017 | Wrong design (guidelines) |
| Dale 2014 | Wrong P (not PANS/PANDAS) |
| Demesh 2015 | Wrong I (tonsillectomy) |
| Ebrahimi 2015 | Wrong I (no treatment) |
| Farhood 2016 | Wrong design (systematic review) |
| Farmer 2018 | Wrong I (validation study) |
| Frankovich | Wrong I (no treatment) |
| Frankovich 2017 | Wrong design (guidelines) |
| Frankovich 2018 | No C (outcome not complications) |
| Gadian 2017 | Wrong design (systematic review) |
| Gagliano 2020 | I missing (data mining for symptoms within the working criteria of PANS) |
| Gilbert 2019 | Wrong design (review) |
| Gromark 2019 | Wrong I (no treatment) |
| Heavey 2019 | Wrong design (review) |
| Hesselmark 2019 | Wrong P (mix of suspected and confirmed PANS), no C |
| Hesselmark 2019 | Wrong I (no treatment) |
| Johnson 2019 | Wrong I (no intervention), no C |
| Latimer 2015 | No C (too few patients for a case series) |
| Leon 2018 | No C (too few patients for a case series) |
| Leonard 2001 | Wrong design (literature review) |
| Lepri 2019 | No C (no systematic information about complications) |

| Maltsev 2020 | Wrong P (autism spectrum disorder and/or cerebral palsy) |
| --- | --- |
| McClelland 2015 | Wrong design (qualitative study) |
| Murphy 2002 | No C (too few patients for a case series) |
| Murphy 2012 | Wrong I (no treatment) |
| Murphy 2013 | Wrong I (tonsillectomy) |
| Murphy 2015 | Wrong P (OCD and/or tics) |
| Nadeau 2015 | No C (too few patients for a case series) |
| Nielsen 2019 | Wrong I (no treatment) |
| Pavone 2014 | Wrong I (tonsillectomy) |
| Perrin 2004 | Wrong/unclear P (group A beta-hemolytic streptococcal infection and risk of PANDAS) |
| Rosa 2018 | Wrong I (food) |
| Sigra 2018 | Wrong design (systematic review) |
| Silverman 2019 | Wrong I (no treatment) |
| Sorensen 2018 | Wrong design (review) |
| Spartz 2017 | No C (outcome not complications) |
| Stingl 2018 | Wrong design (review) |
| Swedo 1998 | Wrong I (no treatment) |
| Swedo 2015 | Wrong I (no treatment) |
| Swedo 2017 | Wrong design (review) |
| Thienemann 2017 | Wrong design (guidelines) |
| Toufexis 2015 | Wrong I (no treatment) |
| Wilbur 2019 | Wrong design (review) |
| Vitaliti 2015 | Wrong design (review) |
| Zheng 2020 | I missing (measure brain structures with MRI in patients corresponding to the PANS criteria and controls) |

^1-52^

**Articles excluded after full-text reading, full reference**

1. Bejerot S, Bruno K, Gerland G, Lindquist L, Nordin V, Pelling H, et al. Suspect PANDAS in children with acute neuropsychiatric symptoms. Infection behind the disease - long-term antibiotic therapy should be considered. Lakartidningen. 2013;110(41):1803-6.
2. Blackburn JS. Tic Disorders and PANDAS. Seminars in Pediatric Neurology. 2018;25:25-33.
3. Burchi E, Pallanti S. Antibiotics for PANDAS? Limited Evidence: Review and Putative Mechanisms of Action. Primary Care Companion to CNS Disorders. 2018;20(3).
4. Calaprice D, Tona J, Murphy TK. Treatment of Pediatric Acute-Onset Neuropsychiatric Disorder in a Large Survey Population. J Child Adolesc Psychopharmacol. 2018;28(2):92-103.
5. Calaprice D, Tona J, Parker-Athill EC, Murphy TK. A Survey of Pediatric Acute-Onset Neuropsychiatric Syndrome Characteristics and Course. Journal of Child and Adolescent Psychopharmacology. 2017;27(7):607-18.
6. Celik G, Tas D, Tahiroglu A, Avci A, Yuksel B, Cam P. Vitamin D Deficiency in Obsessive-Compulsive Disorder Patients with Pediatric Autoimmune Neuropsychiatric Disorders Associated with Streptococcal Infections: A Case Control Study. Noro Psikiyatr Ars. 2016;53(1):33-7.
7. Cocuzza S, Marino S, Gulino A, Pustorino E, Murabito P, Maniaci A, et al. ENT involvement and orobuccal movements' disorders in Pandas patients: assessment and rehabilitations tools. European Review for Medical and Pharmacological Sciences. 2019;23(10):4110-7.
8. Cooperstock MS, Swedo SE, Pasternack MS, Murphy TK. Clinical management of pediatric acute-onset neuropsychiatric syndrome: Part III-Treatment and prevention of infections. J Child Adolesc Psychopharmacol. 2017;27(7):594-606.
9. Dale RC, Brilot F, Duffy LV, Twilt M, Waldman AT, Narula S, et al. Utility and safety of rituximab in pediatric autoimmune and inflammatory CNS disease. Neurology. 2014;83(2):142-50.
10. Demesh D, Virbalas JM, Bent JP. The role of tonsillectomy in the treatment of pediatric autoimmune neuropsychiatric disorders associated with streptococcal infections (PANDAS). JAMA Otolaryngol Head Neck Surg. 2015;141(3):272-5.
11. Ebrahimi Taj F, Noorbakhsh S, Ghavidel Darestani S, Shirazi E, Javadinia S. Group A beta-hemolytic Streptococcal Infection in Children and the Resultant Neuro-psychiatric Disorder; a Cross Sectional Study; Tehran, Iran. Basic Clin Neurosci. 2015;6(1):38-43.
12. Farhood Z, Ong AA, Discolo CM. PANDAS: A systematic review of treatment options. Int J Pediatr Otorhinolaryngol. 2016;89:149-53.
13. Farmer C, Thienemann M, Leibold C, Kamalani G, Sauls B, Frankovich J. Psychometric Evaluation of the Caregiver Burden Inventory in Children and Adolescents With PANS. J Pediatr Psychol. 2018;43(7):749-57.
14. Frankovich J, Leibold CM, Farmer C, Sainani K, Kamalani G, Farhadian B, et al. The Burden of Caring for a Child or Adolescent With Pediatric Acute-Onset Neuropsychiatric Syndrome (PANS): An Observational Longitudinal Study. J Clin Psychiatry. 2018;80(1).
15. Frankovich J, Swedo S, Murphy T, Dale RC, Agalliu D, Williams K, et al. Clinical management of pediatric acute-onset neuropsychiatric syndrome: Part II-use of immunomodulatory therapies. J Child Adolesc Psychopharmacol. 2017;27(7):574-93.
16. Frankovich J, Thienemann M, Pearlstein J, Crable A, Brown K, Chang K. Multidisciplinary clinic dedicated to treating youth with pediatric acute-onset neuropsychiatric syndrome: presenting characteristics of the first 47 consecutive patients. J Child Adolesc Psychopharmacol. 2015;25(1):38-47.
17. Gadian J, Kirk E, Holliday K, Lim M, Absoud M. Systematic review of immunoglobulin use in paediatric neurological and neurodevelopmental disorders. Developmental Medicine and Child Neurology. 2017;59(2):136-44.
18. Gagliano A, Galati C, Ingrassia M, Ciuffo M, Alquino MA, Tanca MG, et al. Pediatric Acute-Onset Neuropsychiatric Syndrome: A Data Mining Approach to a Very Specific Constellation of Clinical Variables. J Child Adolesc Psychopharmacol. 2020.
19. Gilbert DL. Inflammation in Tic Disorders and Obsessive-Compulsive Disorder: Are PANS and PANDAS a Path Forward? Journal of Child Neurology. 2019;34(10):598-611.
20. Gromark C, Harris RA, Wickstrom R, Horne A, Silverberg-Morse M, Serlachius E, et al. Establishing a Pediatric Acute-Onset Neuropsychiatric Syndrome Clinic: Baseline Clinical Features of the Pediatric Acute-Onset Neuropsychiatric Syndrome Cohort at Karolinska Institutet. J Child Adolesc Psychopharmacol. 2019.
21. Heavey E, Peterson K. Treating pediatric acute-onset neuropsychiatric syndrome. Nurse Practitioner. 2019;44(3):44-9.
22. Hesselmark E, Bejerot S. Clinical features of paediatric acute-onset neuropsychiatric syndrome: findings from a case- control study. BJPsych Open. 2019;5(2):e25.
23. Hesselmark E, Bejerot S. Patient Satisfaction and Treatments Offered to Swedish Patients with Suspected Pediatric Acute-Onset Neuropsychiatric Syndrome and Pediatric Autoimmune Neuropsychiatric Disorders Associated with Streptococcal Infections. J Child Adolesc Psychopharmacol. 2019.
24. Johnson M, Fernell E, Preda I, Wallin L, Fasth A, Gillberg C, et al. Paediatric acute-onset neuropsychiatric syndrome in children and adolescents: an observational cohort study. Lancet Child Adolesc Health. 2019;3(3):175-80.
25. Latimer ME, L'Etoile N, Seidlitz J, Swedo SE. Therapeutic plasma apheresis as a treatment for 35 severely ill children and adolescents with pediatric autoimmune neuropsychiatric disorders associated with streptococcal infections. Journal of Child and Adolescent Psychopharmacology. 2015;25(1):70-5.
26. Leon J, Hommer R, Grant P, Farmer C, D'Souza P, Kessler R, et al. Longitudinal outcomes of children with pediatric autoimmune neuropsychiatric disorder associated with streptococcal infections (PANDAS). Eur Child Adolesc Psychiatry. 2018;27(5):637-43.
27. Leonard HL, Swedo SE. Paediatric autoimmune neuropsychiatric disorders associated with streptococcal infection (PANDAS). International Journal of Neuropsychopharmacology. 2001;4(2):191-8.
28. Lepri G, Rigante D, Bellando Randone S, Meini A, Ferrari A, Tarantino G, et al. Clinical-Serological Characterization and Treatment Outcome of a Large Cohort of Italian Children with Pediatric Autoimmune Neuropsychiatric Disorder Associated with Streptococcal Infection and Pediatric Acute Neuropsychiatric Syndrome. J Child Adolesc Psychopharmacol. 2019.
29. Maltsev DV. Efficiency of a High-dose Intravenous Immunoglobulin Therapy in Children with Autism Spectrum Disorders Associated with Genetic Deficiency of Folate Cycle Enzymes. Journal of Global Pharma Technology. 2019;11(5 Supplement):597-609.
30. McClelland M, Crombez MM, Crombez C, Wenz C, Lisius M, Mattia A, et al. Implications for Advanced Practice Nurses When Pediatric Autoimmune Neuropsychiatric Disorders Associated With Streptococcal Infections (PANDAS) Is Suspected: A Qualitative Study. J Pediatr Health Care. 2015;29(5):442-52.
31. Murphy ML, Pichichero ME. Prospective identification and treatment of children with pediatric autoimmune neuropsychiatric disorder associated with group A streptococcal infection (PANDAS). Archives of Pediatrics and Adolescent Medicine. 2002;156(4):356-61.
32. Murphy TK, Lewin AB, Parker-Athill EC, Storch EA, Mutch PJ. Tonsillectomies and adenoidectomies do not prevent the onset of pediatric autoimmune neuropsychiatric disorder associated with group A streptococcus. Pediatr Infect Dis J. 2013;32(8):834-8.
33. Murphy TK, Parker-Athill EC, Lewin AB, Storch EA, Mutch PJ. Cefdinir for recent-onset pediatric neuropsychiatric disorders: a pilot randomized trial. J Child Adolesc Psychopharmacol. 2015;25(1):57-64.
34. Murphy TK, Storch EA, Lewin AB, Edge PJ, Goodman WK. Clinical factors associated with pediatric autoimmune neuropsychiatric disorders associated with streptococcal infections. Journal of Pediatrics. 2012;160(2):314-9.
35. Nadeau JM, Jordan C, Selles RR, Wu MS, King MA, Patel PD, et al. A pilot trial of cognitive-behavioral therapy augmentation of antibiotic treatment in youth with pediatric acute-onset neuropsychiatric syndrome-related obsessive-compulsive disorder. Journal of Child and Adolescent Psychopharmacology. 2015;25(4):337-43.
36. Nielsen MO, Kohler-Forsberg O, Hjorthoj C, Benros ME, Nordentoft M, Orlovska-Waast S. Streptococcal Infections and Exacerbations in PANDAS: A Systematic Review and Meta-analysis. Pediatric Infectious Disease Journal. 2019;38(2):189-94.
37. Pavone P, Rapisarda V, Serra A, Nicita F, Spalice A, Parano E, et al. Pediatric autoimmune neuropsychiatric disorder associated with group a streptococcal infection: the role of surgical treatment. Int J Immunopathol Pharmacol. 2014;27(3):371-8.
38. Perrin EM, Murphy ML, Casey JR, Pichichero ME, Runyan DK, Miller WC, et al. Does group A beta-hemolytic streptococcal infection increase risk for behavioral and neuropsychiatric symptoms in children? Archives of Pediatrics and Adolescent Medicine. 2004;158(9):848-56.
39. Rosa JS, Hernandez JD, Sherr JA, Smith BM, Brown KD, Farhadian B, et al. Allergic Diseases and Immune-Mediated Food Disorders in Pediatric Acute-Onset Neuropsychiatric Syndrome. Pediatr Allergy Immunol Pulmonol. 2018;31(3):158-65.
40. Sigra S, Hesselmark E, Bejerot S. Treatment of PANDAS and PANS: a systematic review. Neurosci Biobehav Rev. 2018;86:51-65.
41. Silverman M, Frankovich J, Nguyen E, Leibold C, Yoon J, Mark Freeman G, Jr., et al. Psychotic symptoms in youth with Pediatric Acute-onset Neuropsychiatric Syndrome (PANS) may reflect syndrome severity and heterogeneity. Journal of Psychiatric Research. 2019;110:93-102.
42. Sorensen CB, Skov L, Lundby L, Grejsen J, Aaslet L, Debes NM. PANDAS and PANS in children and adolescents are still controversial diagnoses. Ugeskrift for Laeger. 2018;180(48).
43. Spartz EJ, Freeman GM, Jr., Brown K, Farhadian B, Thienemann M, Frankovich J. Course of Neuropsychiatric Symptoms After Introduction and Removal of Nonsteroidal Anti-Inflammatory Drugs: A Pediatric Observational Study. J Child Adolesc Psychopharmacol. 2017;27(7):652-9.
44. Stingl C, Cardinale K, Van Mater H. An Update on the Treatment of Pediatric Autoimmune Encephalitis. Curr Treatm Opt Rheumatol. 2018;4(1):14-28.
45. Swedo SE, Frankovich J, Murphy TK. Overview of Treatment of Pediatric Acute-Onset Neuropsychiatric Syndrome. J Child Adolesc Psychopharmacol. 2017;27(7):562-5.
46. Swedo SE, Leonard HL, Garvey M, Mittleman B, Allen AJ, Perlmutter S, et al. Pediatric autoimmune neuropsychiatric disorders associated with streptococcal infections: clinical description of the first 50 cases. Am J Psychiatry. 1998;155(2):264-71.
47. Swedo SE, Seidlitz J, Kovacevic M, Latimer ME, Hommer R, Lougee L, et al. Clinical presentation of pediatric autoimmune neuropsychiatric disorders associated with streptococcal infections in research and community settings. Journal of Child and Adolescent Psychopharmacology. 2015;25(1):26-30.
48. Thienemann M, Murphy T, Leckman J, Shaw R, Williams K, Kapphahn C, et al. Clinical Management of Pediatric Acute-Onset Neuropsychiatric Syndrome: Part I-Psychiatric and Behavioral Interventions. J Child Adolesc Psychopharmacol. 2017;27(7):566-73.
49. Toufexis MD, Hommer R, Gerardi DM, Grant P, Rothschild L, D'Souza P, et al. Disordered eating and food restrictions in children with PANDAS/PANS. Journal of Child and Adolescent Psychopharmacology. 2015;25(1):48-56.
50. Wilbur C, Bitnun A, Kronenberg S, Laxer RM, Levy DM, Logan WJ, et al. PANDAS/PANS in childhood: Controversies and evidence. Paediatrics & Child Health. 2019;24(2):85-91.
51. Vitaliti G, Tabatabaie O, Matin N, Ledda C, Pavone P, Lubrano R, et al. The usefulness of immunotherapy in pediatric neurodegenerative disorders: A systematic review of literature data. Human Vaccines & Immunotherapeutics. 2015;11(12):2749-63.
52. Zheng J, Frankovich J, McKenna ES, Rowe NC, MacEachern SJ, Ng NN, et al. Association of Pediatric Acute-Onset Neuropsychiatric Syndrome With Microstructural Differences in Brain Regions Detected via Diffusion-Weighted Magnetic Resonance Imaging. JAMA Netw Open. 2020;3(5):e204063.
